# Polymer Dispersed Liquid Crystal Imprinted by Microlens Array for Enhanced Outcoupling Efficiency of Organic Light Emitting Diode

**DOI:** 10.3390/molecules29010073

**Published:** 2023-12-22

**Authors:** Seongmin Lim, Hyeon-Sik Ahn, Eun-Jeong Jang, So-Young Boo, Akpeko Gasonoo, Jin-Seog Gwag, Jae-Hyun Lee, Yoonseuk Choi

**Affiliations:** 1 Department of Electronic Engineering, Hanbat National University, Daejeon 34158, Republic of Korea; seongmin625@naver.com (S.L.); princass123@naver.com (H.-S.A.); enjeong722@gmail.com (E.-J.J.); 2 Department of Creative Convergence Engineering, Hanbat National University, Daejeon 34158, Republic of Korea; mick0327@naver.com (S.-Y.B.); jhyunlee@hanbat.ac.kr (J.-H.L.); 3 Department of Chemistry, University of Calgary, Calgary, AB T2N 1N4, Canada; amoebatheo2009@gmail.com; 4 Department of Physics, Yeungnam University, Gyeongsan 38541, Republic of Korea; sweat3000@ynu.ac.kr

**Keywords:** polymer dispersed liquid crystal, outcoupling efficiency, organic light emitting diode

## Abstract

In this paper, we demonstrate the use of polymer dispersed liquid crystal (PDLC) imprinted with a microlens array (MLA) via solution process to improve the outcoupling efficiency of organic light emitting diodes (OLEDs). The PDLC, well known for its scattering effect, is an excellent technology for improving the outcoupling efficiency of OLEDs. Additionally, we introduce a simple spin-coating process to fabricate PDLC which is adaptable for future solution-processed OLEDs. The MLA-imprinted PDLC applied OLED shows an enhancement factor of 1.22 in outcoupling efficiency which is a 37.5% increase compared to the existing PDLC techniques without changing the electrical properties of the OLED. Through this approach, we can expect the roll-to-roll based extremely flexible OLED, and with further research on pattering PDLC by various templates, higher outcoupling efficiency is achievable through a simple UV irradiation process.

## 1. Introduction

Organic light emitting diodes (OLEDs) are flexible electronic devices renowned for their thinness, compact size, and bendability, along with exceptional color reproduction, making them prevalent in today’s display industry [1,2]. OLEDs consume less power compared to light emitting diodes and liquid crystal display technologies. Due to their rapid response time and superior contrast ratio, they are being extensively researched as a next generation display technology [3,4]. An OLED comprises multiple layers of organic materials stacked between two electrodes, the anode and the cathode situated above and below the substrate. However, a significant amount of the light emitted is absorbed at the interface between the OLED device and the air, as well as within the multi-layer organic material due to total internal reflection caused by the large critical angle stemming from the refractive index disparity between layers. Consequently, only about 20% of the light is emitted externally, leading to low efficiency [5,6,7]. To combat this, numerous studies aim to enhance the external light extraction efficiency of OLED devices to improve power efficiency and extend their lifespan [8,9]. Light extraction technologies primarily focus on minimizing light loss by reducing the refractive index mismatch within the OLED, thereby lessening total internal reflection [10,11]. Various optical structures have been integrated into OLED devices for this purpose, including microlens array (MLA) [12,13], visible parylene film [14], MLA patterned parylene substrate [15], random surfaces [16], porous cellulose paper [17], and light scattering layers [18,19,20]. Among these studies, research using parylene has produced a light extraction film that can serve as a flexible substrate and exhibits a high outcoupling efficiency exceeding 20%. However, the drawback is that this film is manufactured using a high temperature and vacuum based chemical vapor deposition (CVD) process, necessitating the establishment of a specific process environment, and resulting in long production times and high costs. Research involving random-surface-based light extraction relies on plasma and gas, while cellulose-based research requires chemical treatments like potassium hydroxide (KOH) and sodium chloride (NaCl), along with extended chemical reaction times for cellulose purification. In both cases, the time required for light extraction film production is lengthy, and the process is complex. In studies using a light scattering layer, a light extraction film using PDLC has been created, but it cannot be applied to flexible OLEDs because it utilizes two glass substrates. Consequently, there is a need for an external light extraction film process with a simplified production environment and process, capable of significantly reducing production time and cost, and suitable for application to flexible OLEDs. Traditional external PDLC light extraction films, which have been investigated for OLED efficiency, are unsuitable for flexible OLEDs due to their reliance on rigid glass substrates and a thick PDLC layer. When the PDLC carries an MLA pattern, it can further improve the outcoupling efficiency of the OLED. Furthermore, MLA-imprinted PDLC prepared by a solution process is expected to enable the production of large area flexible outcoupling films by adopting roll-to-roll, blade, and bar coating methods.

In this paper, our objective is to enhance the outcoupling efficiency of OLEDs by creating a polymer dispersed liquid crystal (PDLC) imprinted with an MLA pattern using spin coating, a solution process method. The use of PDLC for external light extraction has been a long-studied approach to augment OLED performance [21]. PDLC consists of a polymer matrix with liquid crystal (LC) droplets that scatter incoming light by dispersing liquid crystal droplets of several microns in size throughout the polymer matrix. The fabrication process of the proposed MLA-imprinted PDLC is straightforward, involving only spin coating and UV irradiation without the need for any pretreatment to detach the MLA substrate from the PDLC. MLA-imprinted PDLCs exhibit 41% higher haze than conventional PDLCs and have a better light scattering effect. The MLA-imprinted PDLC applied OLED shows an enhancement factor of 1.22 in outcoupling efficiency which is a 37.5% increase compared to the existing PDLC techniques without changing the electrical properties of OLED [22]. MLA-imprinted PDLC is flexible and has high outcoupling efficiency, which is expected to improve the outcoupling efficiency of OLEDs and be used as a flexible substrate.

## 2. Results and Discussion

This PDLC is a liquid-crystal-based material that has long been studied for its light scattering effect to increase the outcoupling efficiency of OLED [23]. Haze is crucial in achieving this effect: the greater the haze, the more the light scatters. As light emitted from the OLED passes through the PDLC, it scatters in various directions. Subsequently, the refractive index at different interfaces changes, allowing part of the light to be redirected and spread outward, which enhances the outcoupling efficiency [24]. The MLA also contributes to improved outcoupling efficiency by refracting incident light at the air lens interface, altering its path and influencing optical properties such as the light outcoupling effect, intensity, and angular distribution [25]. Our research investigates the enhancement of OLED outcoupling efficiency by combining these two methods. To assess the optical impact of the PDLC imprinted with the MLA pattern, a PDLC layer without a pattern was produced for comparison. The resulting PDLC layer had a thickness of 18 μm, while the planarization layer used to flatten the surface formed a thin film of 36.6 μm.

**Figure 1:**
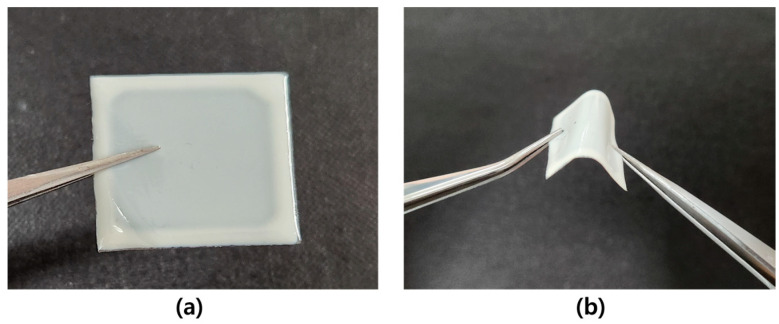
Actual photography of (**a**) MLA-imprinted PDLC; (**b**) bended MLA-imprinted PDLC.

Figure 1 shows an actual photograph of MLA-imprinted PDLC. Figure 1a shows that the PDLC solution has good coating properties, and the edges of the film are where the solution was subjected to surface tension during spin coating. Figure 1b shows the MLA-imprinted PDLC when bent. MLA-imprinted PDLC is flexible and resilient, so it can be expected to be used as a substrate for flexible OLED.

**Figure 2:**
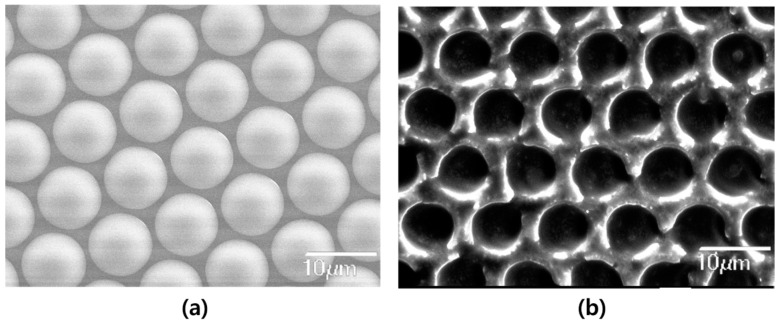
Scanning electron microscopy (SEM) images of: (**a**) the MLA substrate; (**b**) the MLA-imprinted PDLC.

Surface analysis was conducted using SEM to verify the successful imprint of the MLA pattern on the PDLC. Figure 2a shows the pattern of the MLA substrate used, while Figure 2b shows the PDLC with the MLA pattern imprinted after being peeled from the MLA. The MLA substrate utilized had a diameter of 20 μm and a height of 6 μm, and the MLA-imprinted PDLC replicated these dimensions with a diameter of 20 μm but a reduced height of 4 μm. The 2 μm discrepancy in height is attributed to the high viscosity of the NOA 63 solution, which has a viscosity of 2000 cps, preventing the PDLC solution from fully penetrating between the MLA patterns, though the diameter remained consistent. To address this, using a polymer matrix with lower viscosity or heating the solution before coating may aid in the pattern imprinting. The effectiveness of the MLA patterning process was confirmed as it maintained excellent pattern formation even after the peeling process, indicating that the solution process method can achieve impressive MLA patterns, akin to those produced by the deposition process.

**Figure 3:**
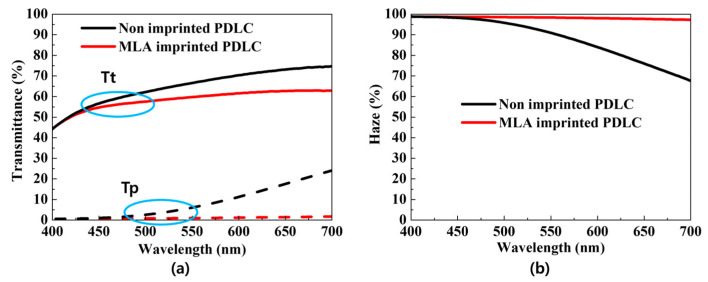
(**a**) Total transmittance and parallel transmittance; (**b**) haze of PDLC without MLA imprint and MLA-imprinted PDLC.

Optical measurements were taken to assess the optical properties of the two types of PDLC. Figure 3a indicates the total transmittance (Tt) and parallel transmittance (Tp) of both the MLA-imprinted and non-imprinted PDLC, while Figure 3b indicates the haze for each type of PDLC. In Figure 3a, a decrease in transmittance with wavelength is observed, which is due to the change in refractive index with the frequency of light, resulting in wavelength-dependent light scattering or absorption. Consequently, as depicted in Figure 3b, the haze increases with shorter wavelengths. At a wavelength of 530 nm, the MLA-imprinted PDLC exhibited a Tt of 58.9% and a Tp of 0.9%, whereas the non-imprinted PDLC showed a Tt of 65% and a Tp of 4.5%. The lower Tp of the MLA-imprinted PDLC indicates a relatively higher light scattering effect compared to the non-imprinted PDLC. When incident light is scattered, Tp decreases and haze increases. The haze value was calculated using a formula that combines Tt and Tp [26]. At 530 nm, the non-imprinted PDLC had a haze of 93%, and the MLA-imprinted PDLC had a haze of 98.4%. With its higher haze value, the MLA-imprinted PDLC has the potential to enhance the outcoupling efficiency of OLEDs. Therefore, the efficiency improvement of OLEDs using MLA-imprinted PDLC was investigated.

**Figure 4:**
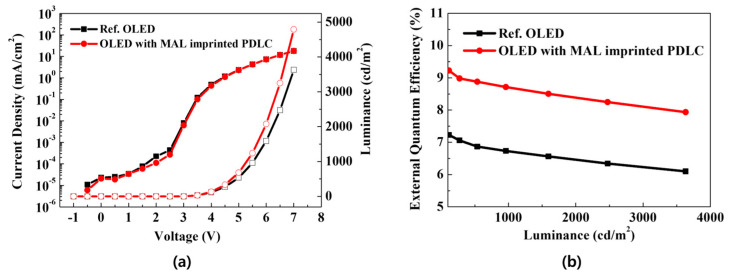
(**a**) Current density −voltage −luminance (J − V − L); (**b**) external quantum efficiency (EQE) of reference OLED and OLED with MLA-imprinted PDLC film.

Figure 4 indicates the electrical and optical characteristics of the reference OLED and the OLED with MLA-imprinted PDLC. The solid symbol is current density-voltage, empty symbol is voltage-luminance in Figure 4a indicates the J-V-L characteristics of both the reference OLED and the OLED with MLA-imprinted PDLC, measured from 1 to 6 V. Both devices were activated at 3 V and exhibited similar J-V characteristics; however, the MLA-imprinted PDLC achieved higher luminance at the same voltage. The reference OLED reached a peak of 3632 cd/m^2^, while the MLA-imprinted PDLC attained a maximum of 4792 cd/m^2^, indicating an increase in luminance efficiency. Figure 4b indicates the EQE of each device. EQE is calculated by multiplying the internal quantum efficiency by the outcoupling efficiency, which allows for an analysis of the ratio of generated photons to injected electrons. As depicted in the EQE graph, a higher luminous efficiency is achieved relative to the number of electrons injected. The reference OLED had a maximum EQE of 6.1%, and this increased to 7.9% when the MLA-imprinted PDLC was applied, indicating an enhancement in EQE. Therefore, it can be concluded that the MLA-imprinted PDLC does not impair the electrical characteristics of the OLED. Instead, it offers improved luminance and power efficiency relative to the reference OLED driving voltage. Additionally, due to its superior luminous performance compared to current efficiency, the MLA-imprinted PDLC is anticipated to be a viable candidate for use as an external light extraction film for OLED.

**Figure 5:**
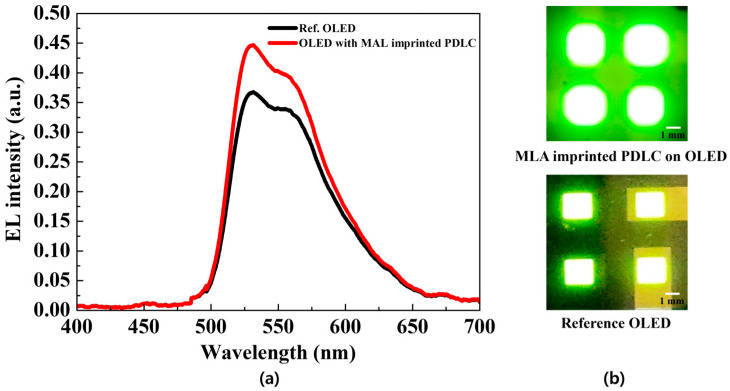
(**a**) Electroluminescence spectrum; (**b**) photographs of OLED with MLA-imprinted PDLC (**top**) and reference OLED (**bottom**).

The analysis of the impact of applying MLA-imprinted PDLC to OLEDs on outcoupling efficiency is presented in Figure 5. Figure 5a indicates the electroluminescence spectrum (EL) of the reference OLED and the OLED with MLA-imprinted PDLC operating at 5 V. It can also be seen that there is a double peak. This is because the phosphorescent dopant, bis(2-phenylpyridine)(acetylacetonate)iridum(Ⅲ) (Ir(ppy)_2_(acac)), consists of iridium, the central atom of which is a heavy atom. The heavy atom effect and the vibronic effect play an important role in controlling the properties of light emitting devices. One of the phenomena caused by these two effects is shoulder peek [27]. Figure 5b indicates a photograph comparing an OLED with the reference OLED and one with MLA-imprinted PDLC applied. A pixel with a size of 4 mm^2^ was produced on the electrode. The reference OLED exhibited an EL intensity of up to 0.36%, while the OLED with MLA-imprinted PDLC showed an increased EL intensity of up to 0.44%. This analysis of outcoupling efficiency improvement confirms that the MLA-imprinted PDLC has an enhancement factor of 1.22. This enhancement is attributed to the strong scattering effect of the MLA-imprinted PDLC and the reduction in total internal reflection as the incident angle of light is decreased below the critical angle at the interface of the light extraction film and air. In summary, the results confirm that MLA-imprinted PDLC effectively improves the outcoupling efficiency of OLED compared to the existing PDLC.

## 3. Materials and Methods

**Figure 6:**
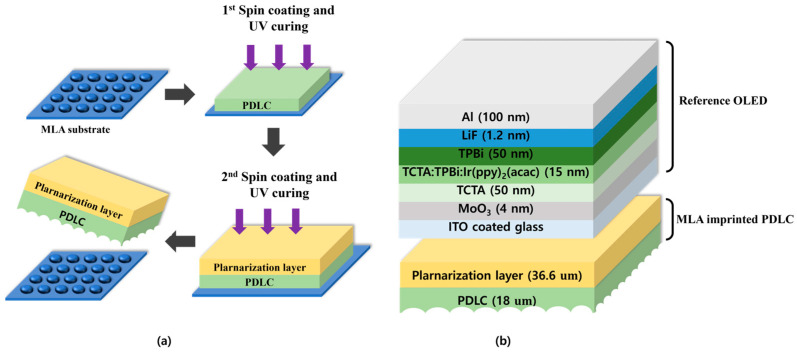
(**a**) Fabrication process of MLA-imprinted PDLC; (**b**) structure of the reference bottom-emitting OLED and the OLED with MLA-imprinted PDLC.

Figure 6a illustrates the manufacturing process of MLA-imprinted PDLC using a spin-coating approach. For the preparation of PDLC solution, nematic liquid crystal (E7, Qingdao QY Liquid Crystal Co., Ltd., Shandong, China) with refractive index *n* = *1.52* and UV curing adhesive (NOA 63, Norland Product, Jamesburg, NJ, USA) a polymer matrix with refractive index *n_p_* = *1.52*, were used to match the refractive index of the used materials to ensure a smooth gap between the OLED and the interface. The difference in refractive index was minimized. This material was mixed at a 40:60 ratio. To ensure smooth mixing of liquid crystal and NOA 63, mixing was carried out at 80 °C, which is the phase transition temperature of liquid crystal.

Next, the MLA substrate was precoated with the PDLC solution at 500 rpm for 5 s and then coated at 1500 rpm for 20 s. UV curing was performed in a UV oven at 150 °C for 15 min at an intensity of 32 mW/cm^2^. This process formed a thin PDLC film on the MLA substrate, resulting in MLA-imprinted PDLC. For films without a pattern, a PDLC thin film was formed under the same conditions on a PET substrate. It is critical to note that the MLA-imprinted PDLC film formed after spin coating is very thin. Post spin coating, surface curvature may occur in the PDLC due to the MLA pattern, leading to an uneven film. If applied to OLED devices in this state, discrepancies in refractive index could produce negative effects. Hence, post imprinting planarization of the film is necessary. NOA 63 is used for its adjustable thickness and transparency, minimizing the impact on OLED light. The planarization spin-coating process involved precoating at 500 rpm for 5 s, followed by coating at 2000 rpm for 20 s. UV curing for 5 min then formed a thin film, which was easily peeled from the MLA substrate without pretreatment, readying a film applicable to OLED.

Figure 6b illustrates the structure of the bottom-emitting reference OLED and the positions where the MLA-imprinted PDLC is applied to comparatively analyze the optical and outcoupling efficiency of the fabricated MLA-imprinted PDLC. The glass substrate, coated with indium tin oxide (ITO, 150 nm), was cleaned by soaking in acetone, isopropyl alcohol, or boiled isopropyl alcohol using an ultrasonic cleaner and then dried in a vacuum oven at 150 °C for 15 min to prepare it for OLED deposition. The OLED organic layers were deposited sequentially as follows: molybdenum oxide (MoO_3_, 4 nm) as a hole injection layer on the precleaned ITO coated glass, 4,4′,4″-tris(carbazol-9-yl)triphenylamine (TCTA, 50 nm) as a hole transport material, bis(2-phenylpyridine)(acetylacetonate)iridium(III) (Ir(ppy)_2_(acac), 15 nm) as a phosphorescent dopant, 1,3,5-tris(N-phenylbenzimidizol-2-yl)benzene (TPBi, 50 nm) as an electron transport layer, and lithium fluoride (LiF, 12 nm) as an electron injection layer. Finally, aluminum (Al, 100 nm) was used as the cathode material.

An alpha-step device (KLA, D-600) was used to measure the thickness of the MLA-imprinted PDLC produced through a solution process using spin coating. Additionally, the surface of the PDLC was analyzed using a scanning electron microscope (SEM, JEOL) to confirm the quality of the MLA imprinting. The optical properties, including transmittance and haze, of the produced MLA-imprinted PDLC and non-imprinted PDLC, were analyzed in the wavelength range of 400 to 700 nm using a UV-visible-NIR spectrometer (PerkinElmer, Lambda 950). To evaluate the efficiency improvement by applying MLA-imprinted PDLC to OLEDs, the current density-voltage-luminance (J-V-L) characteristics and external quantum efficiency (EQE) were measured and analyzed using a source meter (KEITHLEY 2400) and analyzed in the vertical direction using Photo Research (LMS PR 650) software. Voltage measurements were performed in the range of 1 V to 7 V at 0.5 V intervals. The analysis of the outcoupling efficiency before and after the application of MLA-imprinted PDLC was performed using an integrated sphere (IS200-4) and a spectrometer (Thorlabs, CCS200/M).

## 4. Conclusions

In this paper, a PDLC imprinted with an MLA is produced via a solution process to enhance the outcoupling efficiency of OLEDs. This process involving spin coating and UV irradiation can facilitate low-temperature and rather simple fabrication which effectively reduces manufacturing costs and time. Also, this novel PDLC film is flexible and very thin which is an advantage for adopting future full flexible OLEDs without changing any other electrical characteristics of the OLED since it is an external light extraction method. The haze of fabricated PDLC film with MLA pattern was 98.4% which is higher than previous PDLC approaches and resulted in increased outcoupling efficiency. The enhancement factor of outcoupling efficiency with this film was 1.22 which is a 37.5% increase compared to the previous PDLC-based OLED. Furthermore, this PDLC method can vary the spatial optical properties of light extraction layer easily with a simple masking and irradiation process, and it could maximize the outcoupling efficiency of OLEDs with proper manipulation. We believe that this solution-based PDLC film approach can play a critical role in future roll-to-roll processed flexible OLEDs.

## Data Availability

Data are contained within the article.
